# Seroepidemiology of *Toxoplasma gondii* Infection among Healthy Blood Donors in Taiwan

**DOI:** 10.1371/journal.pone.0048139

**Published:** 2012-10-25

**Authors:** Ting-Yi Chiang, Hwei-Ho Hsieh, Ming-Chu Kuo, Kai-Tse Chiu, Wei-Chen Lin, Chia-Kwung Fan, Chi-Tai Fang, Dar-Der Ji

**Affiliations:** 1 Research and Diagnostic Center, Centers for Disease Control, Taipei, Taiwan; 2 Institute of Epidemiology and Preventive Medicine, College of Public Health, National Taiwan University, Taipei, Taiwan; 3 Taipei Blood Center, Taiwan Blood Services Foundation, Taipei, Taiwan; 4 Department of Parasitology, Taipei Medical University College of Medicine, Taipei, Taiwan; 5 Department of Internal Medicine, National Taiwan University Hospital, Taipei, Taiwan; 6 Infectious Diseases Research and Education Center, Department of Health and National Taiwan University, Taipei, Taiwan; 7 Department of Tropical Medicine, National Yang-Ming University, Taipei, Taiwan; Johns Hopkins Bloomberg School of Public Health, United States of America

## Abstract

*Toxoplasma gondii* is an opportunistic, zoonotic pathogen with a worldwide distribution. There are large variations in the seroprevalence of *T. gondii* infection in different regions of the world. Although toxoplasmosis became a notifiable communicable disease in Taiwan in 2007, little is known about its epidemiology among the general population. This cross-sectional study aimed to survey the seroprevalence of *T. gondii* infection and its risk factors among healthy blood donors in Taiwan. Through collaborating with the Taiwan Blood Services Foundation, a total of 1,783 healthy blood donors from all six-branch blood service centers participated in this study. The blood samples were tested for the presence of *T. gondii* antibodies and DNA using enzyme immunoassays and real-time PCR, respectively. Structured questionnaires were used to gather information on risk factors for *T. gondii* infection. Of the 1,783 participants, 166 (9.3%) tested positive for anti-*Toxoplasma* IgG, while 5 (0.28%) tested positive for anti-*Toxoplasma* IgM. The five IgM positive donors had high avidity antibodies suggestive of past infection. No active parasitemia was detected by real-time PCR assays. Multivariate logistic regression showed that undercooked pork meat consumption (adjusted odds ratio [OR] = 2.9; 95% confidence interval [CI]: 1.3–6.5), raw mussels consumption (adjusted OR = 5.3; 95% CI: 1.5–19.1), having a cat in the household (adjusted OR = 2.0; 95% CI: 1.2–3.2), a lower education level (adjusted OR = 1.6; 95% CI: 1.1–2.3), and donation place in eastern Taiwan (adjusted OR = 2.5; 95% CI: 1.6–3.9) were independent risk factors for *Toxoplasma* seropositivity. These findings provide information on the seroprevalence and epidemiology of *T. gondii* infection among healthy blood donors in Taiwan.

## Introduction

*Toxoplasma gondii* is a parasitic protozoan found throughout the world that can be carried by most species of warm blooded animals and infects nearly one-third of the world’s human population [1]–[3]. Although toxoplasmosis occurs worldwide, the seroprevalence of *T. gondii* infection can vary greatly between countries (10–80%) and even within a given country [4]–[5]. *T. gondii* infections in immunocompetent people are usually mild and self-limiting; however, severe disease and complication can occur in immunocompromised individuals and newborns [2], [5]. People typically become infected by three principal routes of transmission: foodborne transmission (consuming undercooked, contaminated meat), animal-to-human transmission (ingesting oocysts shed in the feces of infected cats), and vertical transmission from mother to fetus through the placenta during pregnancy. Additionally, *T. gondii* can be transmitted via blood transfusion or organ transplantation from infected donors [6]–[8].

Toxoplasmosis became a notifiable communicable disease in Taiwan in 2007. Previous studies on the seroprevalence of *T. gondii* antibodies have focused on special groups, such as human immunodeficiency virus (HIV)-infected adults (10.2%) [9], pregnant women (9.1–11.6%) [10]–[11], and mountain aborigines (19.4%–26.7%) [12]–[13]; little is known about the epidemiology of *T. gondii* among the general population. This cross-sectional study aimed to survey the seroprevalence of *T. gondii* infection and associated risk factors among healthy blood donors in Taiwan. We collaborated with the Taiwan Blood Services Foundation, and a total of 1,783 healthy blood donors from all six branch blood service centers participated in this study.

## Materials and Methods

### Study Design

**Figure 1:**
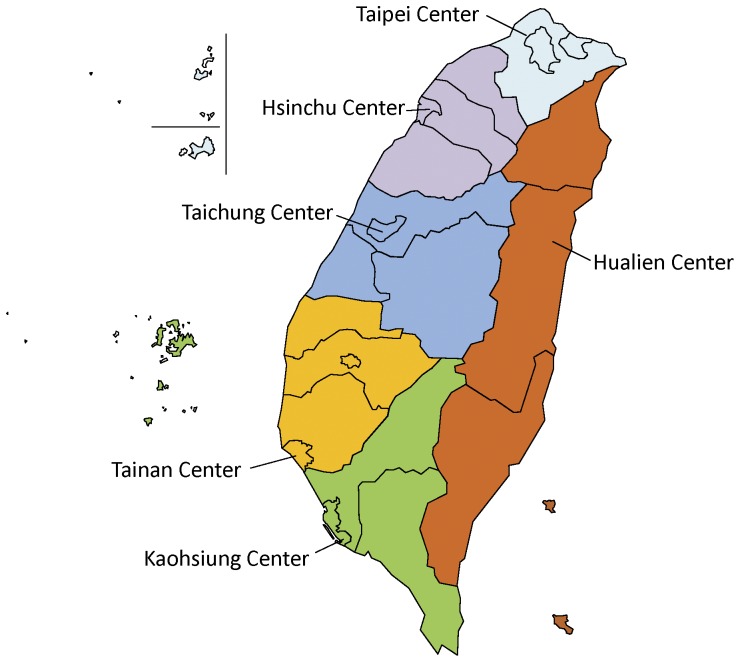
Geographical Distribution of District Blood Service Centers in Taiwan.

We performed a cross-sectional study in all of the six district blood centers of the Taiwan Blood Services Foundation. The six blood centers are located in Taipei (Northern Taiwan), Hsinchu (Northwestern Taiwan), Taichung (Central Taiwan), Tainan (Southwestern Taiwan), Kaohsiung (Southern Taiwan), and Hualien (Eastern Taiwan), respectively (Figure 1).

### Participants

All samples from blood donors were routinely tested for HIV, hepatitis B virus surface antigen (HBsAg), hepatitis C virus (HCV), Human T-Lymphotropic virus (HTLV), and *Treponema pallidum.* Study participants were voluntary blood donors who met the following inclusion criteria: 1) individuals ≥18 years of age; 2) individuals who agreed to participate in the study and gave written informed consent; 3) individuals who tested negative for HIV, HCV, HTLV, *T. pallidum*, and HBsAg.

A total of 1,783 blood samples from healthy blood donors were collected from June 2010 to October 2010. Sample size estimation for each center was based on the proportion of each center’s blood donation from 2008 to 2009 (see Table 1 footnote for details).

**Table 1:** Demographic characteristics of the 1,783 blood donors.

Characteristics	Blood donors No.	Donors with anti-*T. gondii* IgG	
		No.	%
Donation center†			
Taipei	483	44	9.1
Hsinchu	250	21	8.4
Taichung	350	29	8.3
Tainan	250	22	8.8
Kaohsiung	280	18	6.4
Hualien	170	32	18.8
Gender			
Male	1,127	114	10.1
Female	654	52	8.0
Age			
18–25	330	33	10.0
26–35	473	42	8.9
36–45	433	37	8.5
46–55	370	38	10.3
>55	158	16	10.1
Childbearing-aged (18–45 y/o)woman	454	36	7.9
Blood group type			
O	814	74	9.1
A	492	45	9.1
B	331	28	8.5
AB	146	19	13.0
Educational level			
College and above	1,138	88	7.7
High school and below	638	78	12.2
Occupation			
Laborer	332	25	7.5
Businessman/employee	1,061	109	10.3
Student/unemployed	382	32	8.4

† To ensure good representativeness for each region (see Figure 1), sample size estimation for each center was based on the proportion of each center’s blood donation from 2008 to 2009, as listed below:

Taipei (northern Taiwan, regional population: 6,963,205) had 518,696 blood donations in 2008 and 533,399 blood donations in 2009.

Hsinchu (northwestern Taiwan, regional population: 3,462,995) had 253,678 blood donations in 2008 and 243,416 blood donations in 2009.

Taichung (central Taiwan, regional population: 4,479,052) had 366,167 blood donations in 2008 and 365,843 blood donations in 2009.

Tainan (southwestern Taiwan, regional population: 3,429,697) had 276,218 blood donations in 2008 and 290,406 blood donations in 2009.

Kaohsiung (southern Taiwan regional population: 3,749,737) had 311,525 blood donations in 2008 and 313,686 blood donations in 2009.

Hualien (eastern Taiwan, regional population: 1,035,086) had 81,473 blood donations in 2008 and 82,434 blood donations in 2009.

### Ethics

This work was approved by the Institutional Review Board of Taiwan Centers for Disease Control (CDC) (Taipei, Taiwan) and the Institutional Review Board of Taiwan Blood Donor Services Foundation. Written informed consent was obtained from all participants before blood sampling.

### Serologic Testing

Whole blood and serum samples were transported from the six district blood centers to the Parasitic Disease Laboratory of Taiwan CDC (Taipei, Taiwan) and stored at −20°C until analyzed. Serum samples of blood donors were tested for anti-*T. gondii* IgG and IgM antibodies by commercially available enzyme immunoassays (bioMérieux, Marcy l’Etoile, France) with an automated Vitek Immuno Diagnostic Assay System (VIDAS). Analyses were performed as instructed by the manufacturers. For the IgG and IgM assays, positive results were defined as values of ≥8 international units (IU)/ml and index values of ≥0.65. Equivocal results range from 4 to 8 IU/ml and index values of 0.55 to 0.65. Negative results were defined as <4 IU/ml and index values of <0.55. In addition, all IgG-positive/IgM-positive sera were tested using the IgG avidity assay (bioMérieux, Marcy l’Etoile, France). Using the toxoplasmosis IgG avidity assay, low IgG avidity was defined as an index value <0.200, equivocal IgG avidity was defined as 0.200≤ index <0.300, and high IgG avidity was defined as an index value ≥0.3. A high avidity test result using the IgG avidity assay excludes a recently acquired infection within 4 months of serum sampling. The blood group of each donor was confirmed using reverse blood grouping.

### Real-time Polymerase Chain Reaction (PCR)

DNA from the whole blood samples was extracted using the QIAamp DNA Mini Kit (QIAGEN, Valencia, California, USA), according to the manufacturer’s protocol. A real-time PCR assay targeting the 529-bp repeat element (RE) of *T. gondii* was performed as published previously [14], with some modification. The cycling parameters were as follows: preheat at 95°C for 10 min, followed by 45 cycles at 95°C for 15 seconds, 55°C for 30 seconds, and 72°C for 15 seconds. Amplification was carried out with the CFX96 real-time PCR detection system (Bio-Rad Laboratories, Hercules, California, USA). The final reaction mix contained 12.5 µl 2× KAPA PROBE FAST qPCR Master Mix (KAPA Biosystems, Boston, USA), 400 nM of each primer, 800 nM of TaqMan probe, and 5 µl of template DNA in a 25 µl reaction volume. A sample was considered to be positive if the cycle threshold (Ct) value was <40.

### Questionnaire

The questionnaire surveyed basic demographic data, including age, gender, education, residence, and occupation. Possible risk factors, including the sources of drinking water (running water, valley water, ground water, and bottled water), raw/undercooked meat (fish, lamb, beef, pork, oyster, clams, and mussels) consumption, raw vegetables consumption, animal contacts (cats, dogs), gardening or agriculture, prior blood transfusion, and living abroad, were also surveyed.

### Statistical Analysis

The statistical analysis was performed using SPSS 18.0 software package (IBM, Armonk, New York, USA). P values less than 0.05 were considered statistically significant. Logistic regression was used to analyze the association between *T. gondii* seropositivity and potential risk factors. Multivariate logistic analysis was performed with the full model, including all potential risk factors in the analyses.

## Results

### Participants

A total of 1,783 healthy blood donors met the inclusion criteria and were enrolled in the study. Demographic characteristics of the 1,783 healthy blood donors are shown in Table 1 and Table S1. The mean age of the participants in this study was 38.1 years (median age 37 years; range 18 to 65 years). Most participants were male, aged 31–45 years, non-laborers (businessmen or employees), and had a college education or above.

### Seroprevalence of Anti-*T. gondii* Antibodies

Of the 1,783 blood donors, 166 (9.3%) tested seropositive for anti-*T. gondii* IgG antibody, 161 (9.0%) donors tested seropositive for only IgG, and 5 (0.28%) tested seropositive for both IgM and IgG. None were positive for IgM antibody alone. The titers of anti-*T. gondii* IgG positive samples ranged from 9 to 685 IU/ml (median titers: 55.5 IU/ml). Five of the anti-*T. gondii* IgG and IgM positive donors had high avidity antibodies suggestive of prior infection.

By geographic region, anti-*T. gondii* IgG-seropositive rates in Taipei, Hsinchu, Taichung, Tainan, Kaohsiung, and Hualien were 9.1%, 8.4%, 8.3%, 8.8%, 6.4%, and 18.8% (Table 1). By gender, the anti-*T. gondii* IgG-seropositive rates were 10.1% and 8.0% in men and women (p = 0.131), respectively. The anti-*T gondii* IgG-seropositive rate was 7.9% among women of childbearing age (18–45 years). There was no significant difference in the prevalence among different age groups (p = 0.788).

### Real Time PCR

A total of 1,783 blood samples were tested using the real-time PCR assays, and all of the blood samples revealed negative results. For comparison, we performed PCR-restriction fragment length polymorphism (PCR-RFLP) analyses of *T. gondii* DNA from the five clinical specimens (from symptomatic toxoplasmosis cases) stored in our laboratory. *GRA6* marker showed that the *T. gondii* strains in two of the cases (P1 and P3) harbor the type I allele (Figure S1).

### Risk Factors of being Anti-*T gondii* IgG-seropositive

In the univariate analysis, six variables were found to be associated with anti-*T gondii* IgG seropositivity, including donation place at Hualien, lower education level, consumption of valley water, consumption of undercooked pork meat, consumption of raw mussels, and having a cat in the household (Table 2). Other demographic and behavioral characteristics of the blood donors did not show an association with *T. gondii* infection. Further analysis using multivariate logistic regression revealed that the consumption of undercooked pork meat, the consumption of raw mussels, having a cat in the household, a lower education level, and donation place in eastern Taiwan were independent risk factors for *Toxoplasma* seropositivity (Table 3).

**Table 2:** Univariate analysis of the variables associated with *T. gondii* seroprevalence.

Variable	Blood donor No.	Seropositivity(%)	Odds Ratio(95% Confidence interval)	p-value
Donation place				
Hualien	170	18.8	2.6 (1.7–3.9)	<0.001*
Non-Hualien	1,613	8.3	1.0	
Gender				
Male	1,127	10.1	1.3 (0.9–1.8)	0.131
Female	654	8.0	1	
Educational level				
High school and below	638	12.2	1.7 (1.2–2.3)	0.002*
College and above	1,138	7.7	1.0	
Blood group type				
O	814	9.1	1.0	
A	492	9.1	1.0 (0.7–1.5)	0.973
B	331	8.5	0.9 (0.6–1.5)	0.734
AB	146	13.0	1.5 (0.9–2.6)	0.142
Valley water consumption				
Yes	229	13.1	1.6 (1.03–2.4)	0.036*
No	1,553	8.8	1.0	
Undercooked beef meat consumption				
Yes	463	9.3	0.99 (0.7–1.4)	0.974
No	1,317	9.3	1.0	
Undercooked pork meat consumption				
Yes	51	21.6	2.8 (1.4–5.6)	0.003*
No	1,728	8.9	1.0	
Raw fish consumption				
Yes	1,258	9.5	1.1 (0.7–1.5)	0.763
No	522	9.0	1.0	
Raw mussels consumption				
Yes	14	28.6	4.0 (1.2–12.8)	0.034*
No	1,762	9.1	1.0	
Uncooked vegetables consumption				
Yes	1,287	9.7	1.2 (0.8–1.7)	0.365
No	493	8.3	1.0	
Cats in the neighborhood				
Yes	451	10.0	1.1 (0. 8–1.6)	0.564
No	1,313	9.1	1.0	
Cat in the household				
Yes	187	14.4	1.8 (1.1–2.8)	0.012*
No	1,593	8.7	1.0	
Gardening or agriculture				
Yes	649	10.9	1.4 (0.98–1.9)	0.069
No	1,128	8.3	1.0	
Blood transfusion				
Yes	181	9.9	1.1 (0.7–1.8)	0.765
No	1,597	9.3	1.0	
Living abroad (>3 month)				
Yes	78	2.6	1.0	
No	1,698	9.5	4.0 (0.98–16.5)	0.054

* Statistically significant.

**Table 3:** Multivariate logistic regression with full model for risk factors of *T. gondii* infection.

Variable	Adjusted odds ratio(95% Confidence interval)	p-value
Gender	1.3 (0.9–1.8)	0.235
Donation place (Hualien vs. Non-Hualien)	2.5 (1.6–3.9)	<0.001*
Education: high school and below	1.6 (1.1–2.3)	0.007*
Blood group type (A vs. O)	1.1 (0.7–1.6)	0.802
Blood group type (B vs. O)	0.9 (0.6–1.5)	0.688
Blood group type (AB vs. O)	1.6 (0.9–2.7)	0.119
Running water consumption	1.2 (0.7–1.8)	0.554
Valley water consumption	1.6 (1.0–2.7)	0.052
Ground water consumption	1.0 (0.4–2.5)	0.984
Bottled water consumption	0.9 (0.6–1.3)	0.465
Undercooked beef meat consumption	0.8 (0.5–1.2)	0.270
Undercooked lambmeat consumption	0.8 (0.3–2.6)	0.759
Undercooked pork meat consumption	2.9 (1.3–6.5)	0.011*
Raw fish consumption	0.9 (0.6–1.3)	0.510
Raw oysters consumption	1.3 (0.8–2.2)	0.290
Raw clams consumption	1.0 (0.5–1.9)	0.971
Raw mussels consumption	5.3 (1.5–19.1)	0.010*
Uncooked vegetables consumption	1.2 (0.8–1.8)	0.404
Cat in the household	2.0 (1.2–3.2)	0.004*
Cats in the neighborhood	1.0 (0.7–1.4)	0.814
Gardening or agriculture	1.2 (0.8–1.7)	0.297
Blood transfusion	1.0 (0.6–1.8)	0.873
Living abroad (>3 month)	0.2 (0.1–1.0)	0.052

* Statistically significant.

## Discussion

This is the first systematic countrywide seroepidemiologic study on *T. gondii* infection among Taiwanese healthy blood donors, with a large sample size of 1,783 subjects to allow for good representativeness. We found an overall prevalence of *T. gondii* antibodies among the 1,783 blood donors of 9.3%. The seroprevalences in donors from northern, northwestern, central, southwestern, southern, and eastern region of Taiwan were 9.1%, 8.4%, 8.3%, 8.8%, 6.4%, and 18.8%, respectively. These results indicate that *T. gondii* infection exists in all major geographical regions in Taiwan, and that the seroprevalence is significantly higher in eastern Taiwan than in other regions.

Among the blood donors in this study, both the presence of a cat in the household and the consumption of undercooked pork meat are significant risk factors for *T. gondii* seropositivity. This indicates that both infection routes, namely the ingestion of oocysts (animal-to-human transmission) and the ingestion of tissue cysts in meat (foodborne transmission), existed in our study group, similar to the infection routes reported in other countries [15]–[18].

Compared to other countries, our overall seroprevalence was similar to the seroprevalence reported in Mexico [19] and Thailand [20]–[21] but lower than that reported in blood donors from countries including Brazil [22], Chile [23], Malaysia [24], India [25], Egypt [26], Czech Republic [27], and New Zealand [28], where seroprevalences varied from 20.3% to 75.0%. The relatively low overall seroprevalence in Taiwan is probably because the majority of the population in Taiwan eats well-cooked food.

In Taiwan, the general population of ethnic Han Chinese seldom consumes raw pork. Nevertheless, most aborigines have the traditional customs of eating the raw meat of wild pigs, rats, and goats. Previous studies indicated that seroprevalences of *T. gondii* ranged from 19.4% to 26.7% among mountain aboriginal people, and that eating raw meat seems to render individuals more susceptible to *T. gondii* infection [12]–[13]. In 2010, a case of neonate with congenital toxoplasmosis was notified to Taiwan CDC. Field investigation found that the aboriginal mother had eaten raw marinated pork at a gestational age of 24 weeks; the raw pork was highly suspected as the transmission source [29]. In the present study, we found that the prevalence of *T. gondii* seropositivity was significantly higher in the donors from eastern Taiwan (adjusted OR = 2.5; 95% CI: 1.6–3.9), a region with a high proportion of aboriginals among population. The donors from eastern Taiwan were also significantly more likely to report undercooked pork meat consumption (10/169 [5.9%] in eastern Taiwan versus 41/1610 [2.5%] in rest of other regions, p-value = 0.012), suggesting a higher proportion of aboriginal people. However, under the regulations of blood donating services, ethnic information could not be obtained. Therefore, we are unable to verify the association of aboriginal ethnicity and *Toxoplasma* seropositivity. The donation place in eastern Taiwan remains an independent significant risk after adjusting for the effect of pork consumption in multivariate analysis. Therefore, genetic composition of the population might also affect the risk of acquiring toxoplasmosis.

In addition to the association between consumption of raw meat and *T. gondii* infection, our study found that the consumption of raw mussels was also associated with seropositivity of *T. gondii*. Previous experimental studies indicated that *T. gondii* oocysts in seawater could survive long-term and remain infective for several months [30]. In the natural environment, viable oocysts can become concentrated by filter feeders and have been found in various species of shellfish [31]–[33]. Environmentally resistant oocysts that are shed in the feces of infected cats and passed through freshwater flow into the marine environment are considered a contamination source of mussels [34]. Studies in the United States also found that the consumption of raw oysters, clams, and mussels was a risk factor of *Toxoplasma* infection [17], [18].

Through analyzing the demographic characteristics of blood donors, our study found that the seropositivity to *T. gondii* decreased with increasing education level, similar to other reports [19]. We did not find a significant correlation between age or gender with *T. gondii* seroprevalence. The seropositive rate (7.9%) among female donors of childbearing age (aged 18–45) was similar to a previous hospital-based survey on pregnant women (9.1–10.2%) [10]–[11]. Thus, up to 92.1% of childbearing women in Taiwan have not yet been infected and could be at risk to acquire *T. gondii* infection. Furthermore, we found that women of childbearing age are more likely to raise cats in the household than older women (61/454 [13%] women of childbearing age versus 14/200 [7%] older women, p-value = 0.017). Our results suggest that, to prevent congenital toxoplasmosis, health education targeted toward women of childbearing age may be required to minimize the risk of animal-to-human transmission during prenatal and gestation periods.

Moreover, blood transfusion is another potential transmission route for *Toxoplasma* infection. Some studies suggest that toxoplasmosis transmitted through blood transfusion could lead to serious clinical consequences in immunocompromised, immunosuppressed patients and multiple blood transfusion recipients [2], [7]. However, routine screening for *T. gondii* in blood and blood products is not mandatory in Taiwan. In our study, five of our blood donors had IgM antibodies against *T. gondii* and a high avidity index. Therefore, cases with an acute phase of infection could be excluded. Furthermore, the absence of parasitemia by real-time PCR within our blood donors might also exclude the possibility of transmission through blood transfusion.

In Caucasian population, the carriers of AB group are the most susceptible to *T. gondii* infection [35]. In our study, Taiwanese people of blood group AB also had a higher *T. gondii* seroprevalence than people of blood group O. However, the difference was not statistically significant (Table 2 and Table 3).

### Conclusion

We found that *T. gondii* infection among tested healthy blood donors, as evidence by positive anti-*Toxoplasmosis* IgG antibodies, exists in all major regions in Taiwan, with an overall seroprevalence of 9.3%. The consumption of raw pork, which is more common in eastern Taiwan, the consumption of raw mussels, and exposure to domestic cats are most likely the major transmission routes of *T. gondii* among the participants in our study.

As compared to previous studies that focused on specific groups and small-scale samples, this study used countrywide survey data to explore the epidemiology of *T. gondii* in Taiwan and could provide important information on the seroprevalence and potential risk factors for *T. gondii* transmission. However, ethnic information and subjects aged less than 18 years old could not be included in the present study due to ethical regulations. Further studies are needed to focus on these under-surveyed groups, and more surveillance efforts are required for this neglected disease.

## Supporting Information

Figure S1 **PCR-restriction fragment length polymorphism (PCR-RFLP) analyses with** ***GRA6*** **marker.** M: DNA molecular marker; P1–P5: patient1 to patient 5; RH88: reference strain from ATCC.
(TIF)

Table S1 **Characteristics of male vs. female blood donors.**
(DOC)

## References

[1] Hill DE, Chirukandoth S, Dubey JP (2005) Biology and epidemiology of *Toxoplasma gondii* in man and animals. Anim Health Res Rev 6: 41–61. 16164008 10.1079/ahr2005100

[2] Montoya JG, Liesenfeld O (2004) Toxoplasmosis. Lancet 363: 1965–1976. 15194258 10.1016/S0140-6736(04)16412-X

[3] Pappas G, Roussos N, Falagas ME (2009) Toxoplasmosis snapshots: global status of *Toxoplasma gondii* seroprevalence and implications for pregnancy and congenital toxoplasmosis. Int J Parasitol 39: 1385–1394. 19433092 10.1016/j.ijpara.2009.04.003

[4] Sukthana Y (2006) Toxoplasmosis: beyond animals to humans. Trends Parasitol 22: 137–142. 16446116 10.1016/j.pt.2006.01.007

[5] Robert-Gangneux F, Darde ML (2012) Epidemiology of and diagnostic strategies for toxoplasmosis. Clin Microbiol Rev 25: 264–296. 22491772 10.1128/CMR.05013-11 PMC3346298

[6] Remington JS, Klein JO (2001) Infectious diseases of the fetus and newborn infant. Philadelphia: Saunders. Xiv.

[7] Siegel SE, Lunde MN, Gelderman AH, Halterman RH, Brown JA, et al (1971) Transmission of toxoplasmosis by leukocyte transfusion. Blood 37: 388–394. 4927414

[8] Derouin F, Pelloux H (2008) Prevention of toxoplasmosis in transplant patients. Clin Microbiol Infect 14: 1089–1101. 19018809 10.1111/j.1469-0691.2008.02091.x

[9] Hung CC, Chen MY, Hsieh SM, Hsiao CF, Sheng WH, et al (2005) Prevalence of *Toxoplasma gondii* infection and incidence of toxoplasma encephalitis in non-haemophiliac HIV-1-infected adults in Taiwan. Int J STD AIDS 16: 302–306. 15899084 10.1258/0956462053654230

[10] Yu JC (1985) [A seroepidemiological study on *Toxoplasma gondii* infection among pregnant women and neonates in Taiwan]. Taiwan Yi Xue Hui Za Zhi 84: 286–295. 3860602

[11] Hu IJ, Chen PC, Su FC, Hsieh CJ, Jeng SF, et al (2006) Perinatal toxoplasmosis, northern Taiwan. Emerg Infect Dis 12: 1460–1461. 17073106 10.3201/eid1209.060033 PMC3294740

[12] Fan CK, Su KE, Chung WC, Tsai YJ, Chiou HY, et al (1998) Seroprevalence of *Toxoplasma gondii* antibodies among Atayal aboriginal people and their hunting dogs in northeastern Taiwan. Jpn J Med Sci Biol 51: 35–42. 10211430 10.7883/yoken1952.51.35

[13] Fan CK, Su KE, Wu GH, Chiou HY (2002) Seroepidemiology of *Toxoplasma gondii* infection among two mountain aboriginal populations and Southeast Asian laborers in Taiwan. J Parasitol 88: 411–414. 12054025 10.1645/0022-3395(2002)088[0411:SOTGIA]2.0.CO;2

[14] Edvinsson B, Lappalainen M, Evengard B (2006) Real-time PCR targeting a 529-bp repeat element for diagnosis of toxoplasmosis. Clin Microbiol Infect 12: 131–136. 16441450 10.1111/j.1469-0691.2005.01332.x

[15] Cook AJ, Gilbert RE, Buffolano W, Zufferey J, Petersen E, et al (2000) Sources of toxoplasma infection in pregnant women: European multicentre case-control study. European Research Network on Congenital Toxoplasmosis. BMJ 321: 142–147. 10894691 10.1136/bmj.321.7254.142 PMC27431

[16] Baril L, Ancelle T, Goulet V, Thulliez P, Tirard-Fleury V, et al (1999) Risk factors for *Toxoplasma* infection in pregnancy: a case-control study in France. Scand J Infect Dis 31: 305–309. 10482062 10.1080/00365549950163626

[17] Jones JL, Kruszon-Moran D, Wilson M, McQuillan G, Navin T, et al (2001) *Toxoplasma gondii* infection in the United States: seroprevalence and risk factors. Am J Epidemiol 154: 357–365. 11495859 10.1093/aje/154.4.357

[18] Jones JL, Dargelas V, Roberts J, Press C, Remington JS, et al (2009) Risk factors for *Toxoplasma gondii* infection in the United States. Clin Infect Dis 49: 878–884. 19663709 10.1086/605433

[19] Alvarado-Esquivel C, Mercado-Suarez MF, Rodriguez-Briones A, Fallad-Torres L, Ayala-Ayala JO, et al (2007) Seroepidemiology of infection with *Toxoplasma gondii* in healthy blood donors of Durango, Mexico. BMC Infect Dis 7: 75. 17629901 10.1186/1471-2334-7-75 PMC1940003

[20] Pinlaor S, Ieamviteevanich K, Pinlaor P, Maleewong W, Pipitgool V (2000) Seroprevalence of specific total immunoglobulin (Ig), IgG and IgM antibodies to *Toxoplasma gondii* in blood donors from Loei Province, Northeast Thailand. Southeast Asian J Trop Med Public Health 31: 123–127. 11023078

[21] Sukthana Y, Chintana T, Supathanapong W, Siripanth C, Lekkla A, et al (2000) Prevalence of toxoplasmosis in selected populations in Thailand. J Trop Med Parasitol 23: 53–58.

[22] Coelho RA, Kobayashi M, Carvalho LB Jr (2003) Prevalence of IgG antibodies specific to *Toxoplasma gondii* among blood donors in Recife, Northeast Brazil. Rev Inst Med Trop Sao Paulo 45: 229–231. 14502353 10.1590/s0036-46652003000400011

[23] Zamorano CG, Contreras MC, Villalobos S, Sandoval L, Salinas P (1999) [Seroepidemiological survey of human toxoplasmosis in Osorno, Region X, Chile, 1998]. Bol Chil Parasitol 54: 33–36. 10488589

[24] Nissapatorn V, Kamarulzaman A, Init I, Tan LH, Rohela M, et al (2002) Seroepidemiology of toxoplasmosis among HIV-infected patients and healthy blood donors. Med J Malaysia 57: 304–310. 12440270

[25] Elhence P, Agarwal P, Prasad KN, Chaudhary RK (2010) Seroprevalence of *Toxoplasma gondii* antibodies in North Indian blood donors: implications for transfusion transmissible toxoplasmosis. Transfus Apher Sci 43: 37–40. 20605111 10.1016/j.transci.2010.05.004

[26] Elsheikha HM, Azab MS, Abousamra NK, Rahbar MH, Elghannam DM, et al (2009) Seroprevalence of and risk factors for *Toxoplasma gondii* antibodies among asymptomatic blood donors in Egypt. Parasitol Res 104: 1471–1476. 19198880 10.1007/s00436-009-1350-z

[27] Svobodova V, Literak I (1998) Prevalence of IgM and IgG antibodies to *Toxoplasma gondii* in blood donors in the Czech Republic. Eur J Epidemiol 14: 803–805. 9928876 10.1023/a:1007589422080

[28] Zarkovic A, McMurray C, Deva N, Ghosh S, Whitley D, et al (2007) Seropositivity rates for *Bartonella henselae*, *Toxocara canis* and *Toxoplasma gondii* in New Zealand blood donors. Clin Experiment Ophthalmol 35: 131–134. 17362453 10.1111/j.1442-9071.2006.01406.x

[29] Chiang TY, Ji DD (2011) Epidemics of toxoplasmosis in Taiwan. Taiwan Epidemiol Bull 27: 353–360.

[30] Lindsay DS, Dubey JP (2009) Long-term survival of *Toxoplasma gondii* sporulated oocysts in seawater. J Parasitol 95: 1019–1020. 20050010 10.1645/GE-1919.1

[31] Esmerini PO, Gennari SM, Pena HF (2010) Analysis of marine bivalve shellfish from the fish market in Santos city, Sao Paulo state, Brazil, for *Toxoplasma gondii* . Vet Parasitol 170: 8–13. 20197214 10.1016/j.vetpar.2010.01.036

[32] Miller MA, Miller WA, Conrad PA, James ER, Melli AC, et al (2008) Type X *Toxoplasma gondii* in a wild mussel and terrestrial carnivores from coastal California: new linkages between terrestrial mammals, runoff and toxoplasmosis of sea otters. Int J Parasitol 38: 1319–1328. 18452923 10.1016/j.ijpara.2008.02.005

[33] Putignani L, Mancinelli L, Del Chierico F, Menichella D, Adlerstein D, et al (2011) Investigation of *Toxoplasma gondii* presence in farmed shellfish by nested-PCR and real-time PCR fluorescent amplicon generation assay (FLAG). Exp Parasitol 127: 409–417. 20920501 10.1016/j.exppara.2010.09.007

[34] Dubey JP (2004) Toxoplasmosis - a waterborne zoonosis. Vet Parasitol 126: 57–72. 15567579 10.1016/j.vetpar.2004.09.005

[35] Kolbekova P, Kourbatova E, Novotna M, Kodym P, Flegr J (2007) New and old risk-factors for *Toxoplasma gondii* infection: prospective cross-sectional study among military personnel in the Czech Republic. Clin Microbiol Infect 13: 1012–1017. 17617185 10.1111/j.1469-0691.2007.01771.x

