# Supplementary figures and images for: Seroepidemiology of Toxoplasma gondii Infection among Healthy Blood Donors in Taiwan
Source: PLoS One. 2012 Oct 25;7(10):e48139. doi: 10.1371/journal.pone.0048139 (PMC3484999; doi:10.1371/journal.pone.0048139)

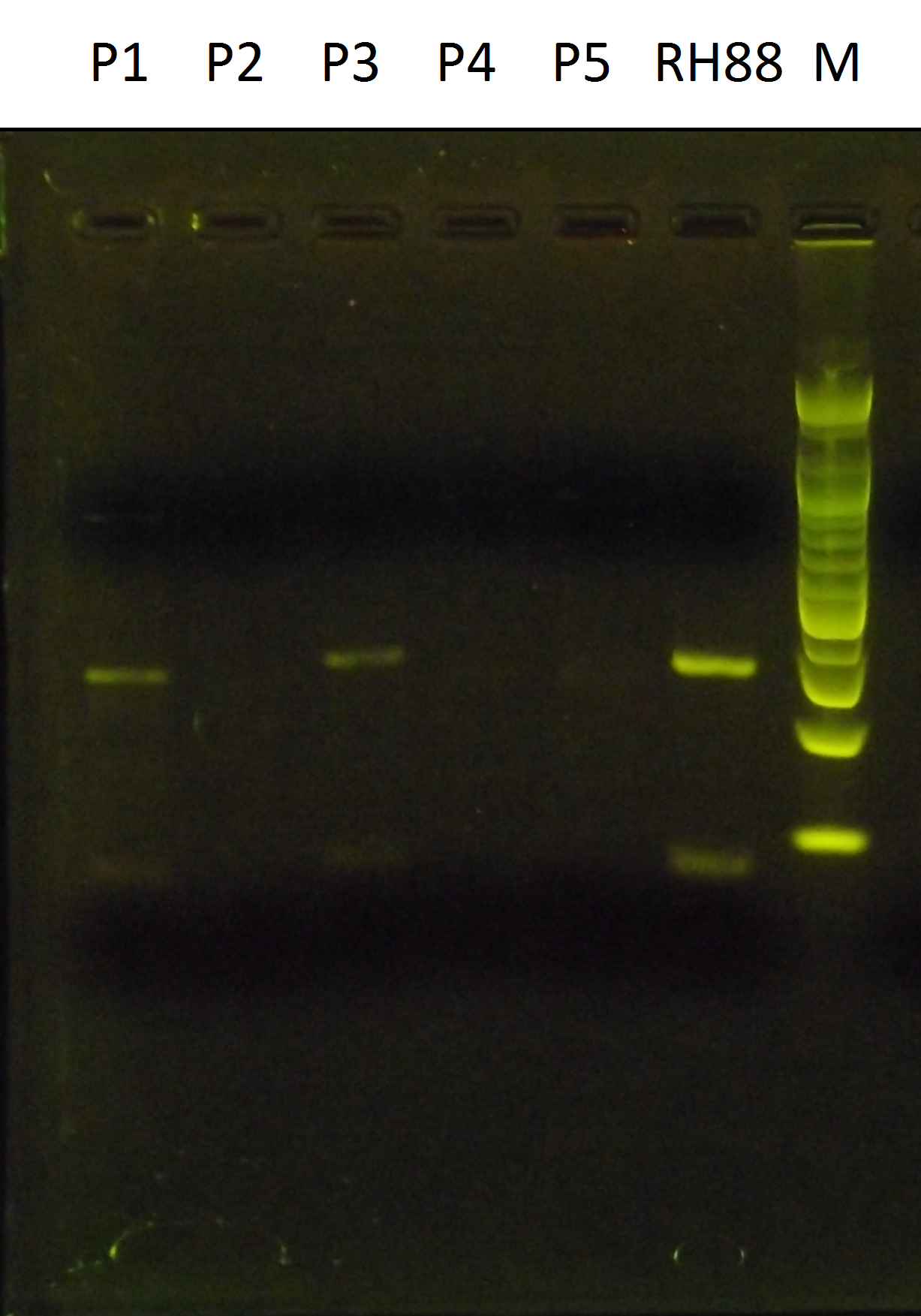

Supplement: Figure S1 — PCR-restriction fragment length polymorphism (PCR-RFLP) analyses with GRA6 marker. M: DNA molecular marker; P1–P5: patient1 to patient 5; RH88: reference strain from ATCC. (TIF) [file pone.0048139.s001.tif]
