# Supplementary material for: Seroepidemiology of Toxoplasma gondii Infection among Healthy Blood Donors in Taiwan
Source: PLoS One. 2012 Oct 25;7(10):e48139. doi: 10.1371/journal.pone.0048139 (PMC3484999; doi:10.1371/journal.pone.0048139)
Supplement: Table S1 — Characteristics of male vs. female blood donors. (DOC) [file pone.0048139.s002.doc]

**Supplementary Table 1**

| **Characteristics** | Blood donors  N (%) | Male  N (%) | Female  N (%) | |
| --- | --- | --- | --- | --- |
| Donation center |  |  | |  |
| Taipei | 483 (27.1) | 278 (24.7) | | 205 (31.3) |
| Hsinchu | 250 (14.0) | 147 (13.0) | | 103 (15.7) |
| Taichung | 350 (19.6) | 236 (20.9) | | 114 (17.4) |
| Tainan | 250 (14.0) | 180 (16.0) | | 70 (10.7) |
| Kaohsiung | 280 (15.7) | 165 (14.6) | | 114 (17.5) |
| Hualien | 170 (9.6) | 121 (10.8) | | 48 (7.4) |
| Age |  |  | |  |
| 18-25 | 330 (18.7) | 170 (15.2) | | 160 (24.8) |
| 26-35 | 473 (26.8) | 313 (28.0) | | 160 (24.8) |
| 36-45 | 433 (24.5) | 298 (26.7) | | 134 (20.8) |
| 46-55 | 370 (21.0) | 237 (21.2) | | 132 (20.5) |
| >55 | 158 (9.0) | 99 (8.9) | | 59 (9.1) |
| Blood group type |  |  | |  |
| O | 814 (45.7) | 502 (44.5) | | 310 (47.4) |
| A | 492 (27.6) | 311 (27.6) | | 181 (27.6) |
| B | 331 (18.5) | 220 (19.5) | | 111 (17.0) |
| AB | 146 (8.2) | 94 (8.4) | | 52 (8.0) |
| Educational level |  |  | |  |
| College and above | 1,138 (64.1) | 735 (65.4) | | 402 (61.8) |
| High school and below | 638 (35.9) | 388 (34.6) | | 249 (38.2) |
| Occupation |  |  | |  |
| Laborer | 332 (18.7) | 274 (24.4) | | 57 (8.8) |
| Businessman/employee | 1,061 (59.8) | 675 (60.0) | | 386 (59.5) |
| Student/unemployed | 382 (21.5) | 175 (15.6) | | 206 (31.7) |
